# Mesenchymal Stem Cells Increase Alveolar Differentiation in Lung Progenitor Organoid Cultures

**DOI:** 10.1038/s41598-019-42819-1

**Published:** 2019-04-23

**Authors:** Kristen T. Leeman, Patrizia Pessina, Joo-Hyeon Lee, Carla F. Kim

**Affiliations:** 1 Department of Pediatrics, Division of Newborn Medicine, Boston Children’s Hospital, Boston, MA 02115 USA; 2 Department of Pediatrics, Division of Hematology/Oncology, Stem Cell Program; Boston Children’s Hospital, Boston, MA 02115 USA; 3 Genetics Department, Harvard Medical School, Boston, MA 02115 USA; 4 Harvard Stem Cell Institute, Cambridge, MA 02138 USA; 5 Wellcome Trust/Medical Research Council Stem Cell Institute, University of Cambridge, Tennis Court Road, Cambridge, CB2 1QR UK

**Keywords:** Mesenchymal stem cells, Multipotent stem cells

## Abstract

Lung epithelial cell damage and dysfunctional repair play a role in the development of lung disease. Effective repair likely requires the normal functioning of alveolar stem/progenitor cells. For example, we have shown in a mouse model of bronchopulmonary dysplasia (BPD) that mesenchymal stem cells (MSC) protect against hyperoxic lung injury at least in part by increasing the number of Epcam^+^ Sca-1^+^ distal lung epithelial cells. These cells are capable of differentiating into both small airway (CCSP^+^) and alveolar (SPC^+^) epithelial cells in three-dimensional (3D) organoid cultures. To further understand the interactions between MSC and distal lung epithelial cells, we added MSC to lung progenitor 3D cultures. MSC stimulated Epcam^+^ Sca-1^+^ derived organoid formation, increased alveolar differentiation and decreased self-renewal. MSC-conditioned media was sufficient to promote alveolar organoid formation, demonstrating that soluble factors secreted by MSC are likely responsible for the response. This work provides strong evidence of a direct effect of MSC-secreted factors on lung progenitor cell differentiation.

## Introduction

Lung diseases have large impacts on both pediatric and adult patients throughout the world. For example, the pathology in bronchopulmonary dysplasia (BPD) is characterized by depletion of or damage to the lung epithelial cells that normally facilitate gas exchange coupled with a disruption in vascular development^1,2^. A critical need for investigation of mechanisms of lung disease and novel therapeutics remains.

Repair in the distal lung is provided by a number of stem/progenitor populations, including Epcam^+^ Sca-1^+^ distal lung epithelial cells (a population enriched in lung progenitors formerly referred to as bronchioalveolar stem cells (BASCs)^3^), club (Clara) cells, AT2 cells, and alveolar progenitors. Lineage tracing studies and injury studies have shown club cells act as stem cells, producing club cells and ciliated cells^4^. AT2 cells and Integrin-alpha-6-expressing progenitors, also known as lineage-negative progenitors, produce AT2 cells and alveolar type 1 (AT1) cells^5–8^. Basal cells have been shown to be proximal airway progenitor cells in mice and are present in human distal airways^9^. Co-expression of the bronchiolar club cell marker, CCSP, and the AT2 cell marker, SPC (pro-surfactant protein C), in Epcam^+^ Sca-1^+^ distal lung epithelial cells (hereafter, Sca-1^+^ cells) and their expansion seen after lung injury *in vivo*, suggested that these cells function in epithelial repair in the bronchiolar and alveolar compartments^3,10^. We and others have developed 3D co-culture systems to test the stem cell properties of lung epithelial cells when cultured with supporting stroma^10–13^. These systems permit alteration of lung stem cell type or stromal cell type to test effects on organoid growth, differentiation and self-renewal properties. These 3D co-culture approaches have revealed that Sca-1^+^ cells produce lung cell organoids containing bronchiolar and alveolar cell types, providing evidence that they are multipotent lung stem cells^10^. While the direct relationships between lung stem cell potential in culture and *in vivo* remain to be determined, these and related findings suggest that many different distal lung cell types have the capacity to respond to lung injury^1,14^. Further, these data support the idea that lung injury repair *in vivo* is dependent on the specific type and location of injury, severity of damage, and the degree to which stroma that signal to epithelial cells are affected.

For progenitor cells to properly repair lung injury, such as damage to alveolar epithelial cells, it is necessary for a signal(s) to instruct the progenitor cell to produce alveolar progeny^15,16^. The precise signals from the microenvironment that stimulate differentiation for repair of lung injury are unknown. Mesenchymal stem cell (MSC) delivery prevents lung injury in multiple animal models, including in the established neonatal hyperoxia mouse model of BPD^17,18^. MSC engraftment in these injury models is minimal and therapeutic benefit is likely triggered by a paracrine-mediated mechanism^19^. Both MSC and MSC- conditioned media (CM) treatment not only protected mice from injury, but also increased lung progenitors number *in vivo*^14,17^. Signals secreted from MSC likely activate lung epithelial progenitor cells to differentiate and repair injury. Microvesicles secreted from the MSC have been proposed to be the therapeutic factor present in MSC-CM for repair of lung injury^20,21^. It is possible that MSC-secreted factors replace missing microenvironmental signals that have been altered by injury to the lung supporting cells. This work uses a robust lung organoid system to show that critical signals secreted by the MSC trigger lung progenitor cells to differentiate and potentially play a critical role in enhancing repair of alveolar injury.

## Results

### Mesenchymal stem cells increase lung organoid formation

**Figure 1:**
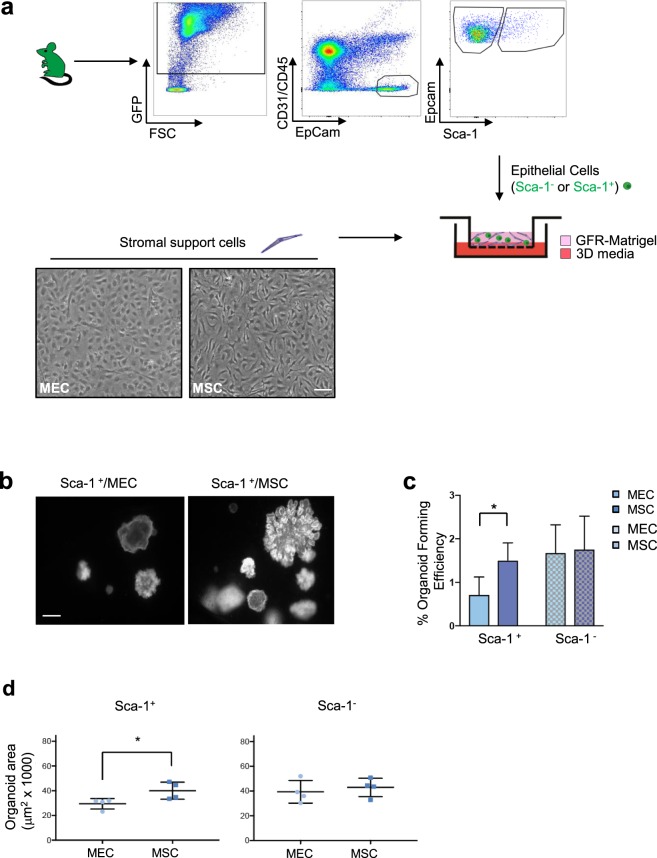
Mesenchymal Stem Cells Increase Lung Organoid Formation in 3D Culture. (**a**) Schematic of FACS strategy and 3D organoid co-culture methods. Fresh lung cells were isolated from β-actin GFP mice and FACS strategy represents signature used to enrich for Epcam^+^ Sca-1^−^ cells and Epcam^+^ Sca-1^+^ cells. CD45^+^ hematopoietic and CD31^+^endothelial cells were first excluded. Epcam^+^ epithelial cells were selected and Sca-1^+^ cells were enriched for lung progenitors and Sca-1^−^ cells were enriched for AT2 cells. Isolated cells were placed in co-culture with either mouse lung endothelial cells (MEC) or mouse bone marrow derived mesenchymal stem cells (MSC) in growth factor reduced matrigel on an air-liquid interface 3D co-culture system. Representative images of the different stromal cells are shown in the lower panel. Scale bar: 50μM. (**b**) Representative images of GFP^+^ organoids formed from 3D co-culture of Sca-1^+^ cells with MEC or MSC after 14 days in co-culture. Scale bar: 100μM (**c**) Organoid forming efficiency (OFE) of Sca1^+^/MEC co-cultures was 0.875 which was significantly decreased compared to Sca1^+^/MSC (1.5 OFE) (p < 0.02). Quantification of number of GFP^+^ lung organoids formed in co-culture after 14 days in culture showed a significant 1.7x increase in total Sca-1^+^ colony number when co-cultured with MSC versus MEC. No significant difference in organoid forming efficiency is observed when Sca-1^−^ cells are cultured with MEC versus MSC, Sca-1^−/^MEC OFE was 1.685 and Sca-1^−^/MSC OFE was 1.76. (**d**) Organoid size measured on GFP^+^ pictures from the indicated co-cultures show an increased colony size with Sca1^+^/MSC co-cultures compared to Sca1^+^/MEC co-cultures, p < 0.05. Data represented is the mean from 4 independent experiments with 3 wells per experimental state in each experiment. Error bars represent SD.

3D co-culture experiments illustrated a direct effect of MSC on Sca-1^+^ cells by exhibiting a significantly increased organoid forming efficiency (Fig. 1). We have previously shown that in the mouse model of BPD, bone-marrow derived MSC (BMSC) treatment led to an increased number of Sca-1^+^ cells *in vivo* and *in vitro* using traditional 2-dimensional cultures^14^. Sca-1^+^ cells and Sca-1^−^ cells (enriched for AT2 cells) were freshly isolated from 6–8 week old β-actin GFP mice or DsRed mice using established FACS signature (Sca-1^+^ distal lung progenitors: DAPI^−^, CD31^−^, CD45^−^, EPCAM^+^, Sca-1^+^; AT2 cells: DAPI^−^, CD31^−^, CD45^−^, EPCAM^+^, Sca-1^−^) (Fig. 1a, S1a). Sca-1^+^ and Sca-1^−^ cells were co-cultured with either mouse derived MSC or lung mouse endothelial cells (MEC) in growth-factor reduced matrigel on an air liquid interface for 14 days (Fig. 1a). MEC were chosen as the comparison stromal population due to previous work establishing their role in lung progenitor cell differentiation^10^. The number of Sca-1^+^ organoids formed on day 14 was significantly increased by 1.7-fold when co-cultured with MSC compared to MEC. Specifically, the organoid forming efficiency (OFE) of Sca-1^+^/MEC co-cultures was 0.875, which was significantly decreased, compared to Sca-1^+^/MSC (1.5 OFE) co-cultures (p < 0.02) (Fig. 1b,c). Sca-1^−^ organoid formation was unaffected by stromal cell modulation between MSC and MEC; 3D cultures showed a nonsignificant difference in organoid forming efficiency with Sca-1^−^/MEC OFE 1.685 and Sca-1^−^/MSC OFE 1.76 (Fig. 1c). These experiments suggested that MSC selectively alter Sca-1^+^ progenitors and do not affect other Sca-1^−^ lung progenitor cells such as AT2 cells. Furthermore, Sca-1^+^-derived organoids are larger when cultured with MSC compared to MEC (1.35-fold p < 0.05) (Fig. 1d), indicating that MSC enhance Sca-1^+^ cell proliferation and this is in agreement with previous *in vivo* results showing increased distal lung progenitors number in a neonatal murine model of BPD combined with mesenchymal stem cells treatment^14^. Sca-1^–^derived organoids showed no significant change in organoid size when co-cultured with MSC compared to MEC.

### Mesenchymal stem cells stimulate alveolar differentiation of Epcam^+^ Sca-1^+^ distal lung epithelial cells

**Figure 2:**
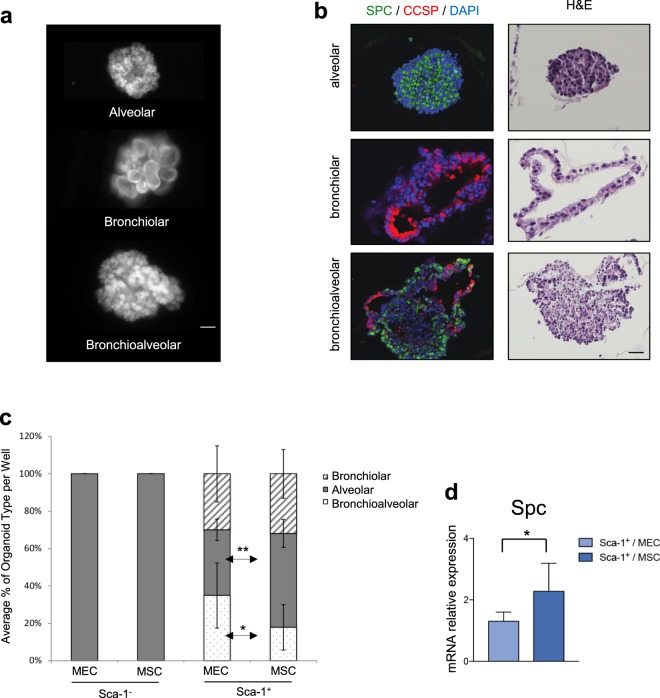
Mesenchymal Stem Cells Increase Distal Lung Epithelial Cell Differentiation into Alveolar Organoids in 3D Culture. (**a**) Representative GFP^+^ images of distinct organoid types formed. Alveolar (top), bronchiolar (middle) and bronchioalveolar/mixed (bottom) organoids from Sca-1^+^ co-cultures. Only alveolar (top) organoids were formed from Sca-1^−^ cell co-cultures. Scale bar: 50 μM. (**b**) Representative Hematoxylin/Eosin (HE) images (right panel) and immunofluorescence staining for SPC (green) and CCSP (red) (left panel) on the three organoid types. Nuclei are stained with DAPI (blue). Scale bar: 50μM. (**c**) Quantification of each colony type from Sca-1^−^ or Sca-1^+^ cell co-cultures with MEC or MSC. Sca-1^−^ cells co-cultured with either MEC or MSC formed 100% alveolar organoids. Sca-1^+^ co-cultured with MEC (Sca-1^+^/MEC cultures) formed 34.7% bronchioalveolar/mixed organoids, 34.8% alveolar organoids, and 29.7% bronchiolar organoids. Sca-1^+^ cells co-cultured with MSC (Sca-1^+^/MSC cultures) formed 17.9% bronchioalveolar/mixed organoids, 50.2% alveolar organoids and 31.9% bronchiolar organoids. Increased differentiation of Sca-1^+^/MSC cultures to alveolar lineage compared to Sca-1^+^/MEC cultures was observed (50.2% versus 34.8%; p = 0.005). Decreased bronchioalveolar/mixed organoids were found in Sca-1^+^/MSC cultures compared to Sca-1^+^/MEC cultures (17.9% versus 34.7%; p = 0.05). 7 independent experiments were performed with 3 wells or greater per experimental state in each experiment, greater than 750 Sca-1^–^and Sca-1^+^- derived organoids analyzed. Error bars represent SD. (**d**) QPCR expression for the alveolar marker *Spc* in Sca-1^+^/MEC and Sca-1^+^/MSC. Data represented is the mean from four independent experiments with triplicate wells per experimental state in each experiment. Error bars represent SD, p < 0.05.

To determine if MSC in Sca-1^+^ co-cultures influenced differentiation, we assessed the efficiency of lineage-specific organoid formation. Overall, Sca-1^+^ co-cultured with MSC produced a distinct differentiation pattern compared to Sca-1^+^ cells co-cultured with MEC. After 14 days in co-culture, alveolar, bronchiolar, and bronchioalveolar (mixed) organoids were identified by morphology and by immunofluorescence staining for alveolar (SPC) and bronchiolar (CCSP) markers (Fig. 2a,b). Sca-1^+^/MSC co-cultures formed a significantly higher number of alveolar organoids compared to Sca-1^+^/MEC co-cultures (50.2% versus 34.8%; p = 0.005). Sca-1^+^/MEC co-cultures fostered the bronchioalveolar (mixed) organoid formation with a higher number of mixed organoids formed compared to Sca-1^+^/MSC co-cultures (34.7% versus 17.9%; p = 0.05). 778 organoids from Sca-1^+^ co-cultures were analyzed (298 organoids from Sca-1^+^ /MEC co-culture and 480 organoids from Sca-1^+^/MSC co-culture). Sca-1^−^ cultures formed 100% alveolar organoids when cultured with either MEC or MSC. 1171 organoids from Sca-1^−^ co-cultures were analyzed (652 organoids from AT2/MEC co-culture and 519 organoids from AT2/MSC co-culture) (Fig. 2c). Confirming this result, qPCR analysis for *Spc* showed increased expression in Sca-1^+^-derived organoids when co-cultured with MSC compared to MEC (Fig. 2d). We also further characterized our alveolar organoids for evidence of alveolar type 1 (AT1) cells. Confirming that AT2 cells differentiate in our culture conditions, we detected cells that stained with antibodies for Aquaporin 5 and Podoplanin, two AT1 cell markers, in alveolar organoids. No significant difference was seen in the fraction of organoids containing AT1 cell markers when co-cultured with MEC versus MSC, supporting that MSC did not cause a change in AT2 to AT1 differentiation (Fig. S1b,c). These data show that lung progenitor differentiation can be altered by stromal signals in co-culture. Specifically, MSC stimulate Sca-1^+^ to form a higher number of differentiated alveolar organoids at the expense of bronchioalveolar organoids.

### Mesenchymal stem cells reduce Sca-1^+^ distal lung epithelial cell self renewal ability

**Figure 3:**
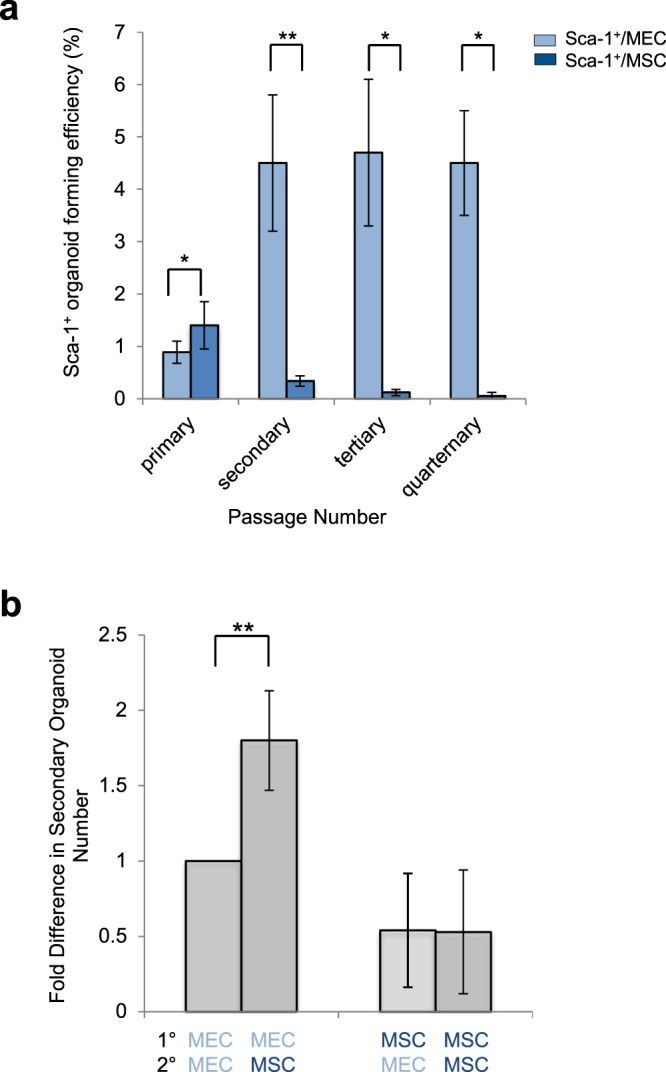
Mesenchymal Stem Cells Decrease Lung Progenitor Self-Renewal Functions. (**a**) Self-renewal of Sca-1^+^ cells in 3D organoid culture was decreased when co-cultures with MSC compared to MEC. Primary organoids were dissociated and GFP^+^ cells were replated for secondary, tertiary and quaternary organoid formation with MEC or MSC. Organoid forming efficiency (OFE) is the number of organoids formed/number of cells plated per well as a percentage. Sca-1^+^ cells serially passaged with MEC retained organoid forming efficiency ranging from 4.5–4.7% while Sca-1^+^ cells serially passaged with MSC lost ability to form organoids in secondary and subsequent passages. Secondary passage Sca-1^+^/MEC cultures had 4.5% OFE versus Sca-1^+^/MSC cultures 0.34%, p = 0.005. Tertiary passage Sca-1^+^/MEC cultures had 4.7% OFE versus Sca-1^+^/MSC cultures 0.12% OFE, p = 0.03. Quaternary passage Sca-1^+^/MEC cultures had 4.5% OFE versus Sca-1^+^/MSC cultures 0.05%, p = 0.03. Data presented are the mean of three independent experiments with triplicate wells. Error bars indicate SD. (**b**) Quantification of organoid formation when stromal cell population type was altered between primary and secondary passage. Sca-1^+^/MEC cultures have a 1.8x fold (p = 0.005) increased organoid formation when co-cultured with MSC in secondary passage compared to MEC. No difference in secondary passaging ability seen in primary Sca-1^+^/MSC cultures. MSC retain ability to enhance Sca-1^+^ derived organoid formation in secondary passage when multi-potent state is fostered in primary passage. Data presented are the mean of three independent experiments with triplicate wells. Error bars indicate SD.

Next, serial passaging with different stromal cell/Sca-1^+^ combinations was used to determine if MSC also impacted self-renewal functions. These experiments revealed that self-renewal was significantly decreased in Sca-1^+^/MSC cultures compared to Sca-1^+^/MEC cultures (Fig. 3a). Sca-1^+^ cells serially passaged with MEC retained organoid forming efficiency ranging from 4.5–4.7%, while their serially passaged with MSC lost ability to form organoids in secondary and subsequent passages; secondary passage Sca-1^+^/MEC 4.5% versus Sca-1^+^/MSC 0.34%, p = 0.005; tertiary passage Sca-1^+^/MEC 4.7% versus Sca-1^+^/MSC 0.12%, p = 0.03; quaternary passage Sca-1^+^/MEC 4.5% versus 0.05%, p = 0.03. (Fig. 3a). MSC retained the ability to enhance Sca-1^+^ organoid formation in secondary passage when the multi-potent state was initially fostered in primary passage with MEC, Sca-1^+^-MEC cultures had a significant 1.8x fold (p = 0.045) increased colony formation when co-cultured with MSC in secondary passage compared to MEC (Fig. 3b). No difference in secondary passaging ability with MEC versus MSC was observed for cells passaged from primary Sca-1^+^/MSC cultures. MEC co-culture did not restore the cells’ ability to form multipotent organoids in serial passage. This finding, together with the observed increase in alveolar organoid formation in Sca-1^+^/MSC co-cultures, supports the interpretation that MSC trigger irreversible lung progenitor differentiation and an associated decrease in self-renewal compared to the self-renewal stimulation seen with MEC.

### Mesenchymal stem cell secreted factors stimulate lung organoid formation

**Figure 4:**
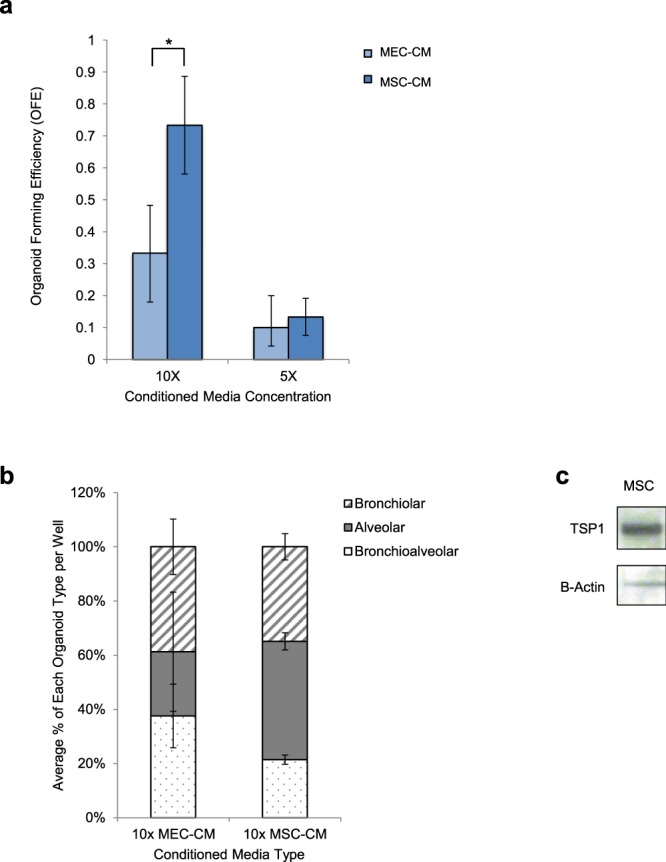
Mesenchymal Stem Cell Conditioned Media Alone Increases Lung Organoid Formation in 3D Culture. (**a**) MSC-secreted factors alone increase organoid number when Sca-1^+^ cells plated with concentrated MEC-conditioned media (CM) or MSC-CM media at 10x or 5x concentration. A significant 2.22x fold increase in total Sca-1^+^ organoid number was found in Sca-1^+^/10x MSC-CM cultures compared to Sca-1^+^ /10x MEC-CM cultures; p = 0.02. A nonsignificant 1.33x fold increase in total Sca-1^+^ organoid number was found in Sca-1^+^/5x MSC-CM cultures compared to Sca-1^+^/5x MEC-CM cultures, p > 0.05. No organoid formation was seen with Sca-1^+^ cells cultured in 1x or 0.1x CM or non-conditioned medium from either stromal cell type (data not shown). Data presented are the mean of three independent experiments with triplicate wells. Error bars indicate SD. (**b**) Quantification of each organoid type from Sca-1^+^ cell co-cultures with 10 x MEC-CM or 10 x MSC-CM. Sca-1^+^ co-cultured with 10x MEC-CM (Sca-1^+^/MEC-CM cultures) formed 37.6% bronchioalveolar/mixed organoids, 23.7% alveolar organoids, and 38.7% bronchiolar organoids. Sca-1^+^ co-cultured with 10x MSC-CM (Sca-1^+^/MSC-CM cultures) formed 21.4% bronchioalveolar/mixed organoids, 42.7% alveolar organoids and 34.8% bronchiolar organoids. A trend towards increased differentiation of Sca-1^+^/MSC-CM cultures to alveolar lineage compared to Sca-1^+^/MEC-CM cultures was observed (42.7% versus 23.7%; p > 0.05) and decreased bronchioalveolar/mixed organoids were found in Sca-1^+^/MSC-CM cultures compared to Sca-1^+^/MEC-CM cultures (21.4% versus 37.6%; p > 0.05) was found but did not reach statistical significance. Data presented are the mean of three independent experiments with triplicate wells. Error bars represent SD. (**c**) Western Blotting Analysis of MSC. A western blot was performed on lysates from MSC for TSP1 and B-actin.

Based on previous work suggesting MSC-secreted factors acting as therapeutic components, we tested the effect of MSC-conditioned media alone on lung stem cell functions in 3D culture. In these experiments, no stromal cell population was present indicating any change in stem cell properties was due to secreted factors alone. When Sca-1^+^ cells were cultured with conditioned media (CM) alone, more organoids formed using MSC-CM compared to MEC-CM (Fig. 4a). Culturing with MSC-CM concentrated 10×, Sca-1^+^ cells formed a 2.22 fold more organoids (p = 0.02) compared to cultures with 10x MEC-CM. MSC-CM concentrated 5x showed a similar trend and yielded 1.33 fold more organoids compared to 5x MEC-CM, but did not reach statistical significance (Fig. 4a). No organoids were formed when 1x CM or 0.1x CM from either MSC or MEC or media alone was used (data not shown). A trend towards increased differentiation of Sca-1^+^/MSC-CM cultures to alveolar lineage compared to Sca-1^+^/MEC-CM cultures was observed (42.7% versus 23.7%; p > 0.05) and decreased bronchioalveolar/mixed organoids were found in Sca-1^+^/MSC-CM cultures compared to Sca-1^+^/MEC-CM cultures (21.4% versus 37.6%; p > 0.05) but did not reach statistical significance (Fig. 4b). These experiments support that MSC secreted factors are responsible for the direct effect that MSCs impart on lung progenitors in organoid cultures.

A semi-quantitative membrane antibody array containing labels corresponding to 308 mouse proteins was used to identify candidate factors produced by MSC that may mediate the effects we observed on organoid formation. MSC were cultured in serum free media and supernatant was collected and was incubated with the membrane array (AAM-BLM-1, Ray-Biotech). Biotin-labeled MSC supernatant was incubated with the array overnight and hybridized with HRP-conjugated streptavidin and results were analyzed. TSP, ICAM, MMP-9, and Epigen were identified on the antibody array and present in the MSC conditioned media (Fig. S2). We have previously shown that TSP1 stimulates alveolar differentiation of lung progenitor cells in organoid cultures and *in vivo*^10^, making TSP1 a strong candidate factor in mediating the MSC effects we observed. Western blot analysis confirmed abundant TSP1 protein in MSCs (Fig. 4c). These experiments support the hypothesis that TSP1 and other MSC-secreted factors are responsible for the direct effect that MSCs impart on lung progenitors in organoid cultures.

## Discussion

MSC-secreted factors have been hypothesized to be therapeutic activators of lung repair based on multiple animal models of lung injury and MSC treatments. MSC–secreted factors may prevent disease by replacing defective signals after injury and thereby triggering endogenous epithelial progenitor cells to repair injured epithelium. Determination of the active soluble factors secreted by MSC remains an active area of investigation and critical next step to understanding this direct effect. This work shows that MSC-secreted factors can act directly upon epithelial progenitor cells to alter their functions compared to MEC. Specifically, while MEC foster lung progenitor self renewal, MSC increase organoid formation and alveolar differentiation compared to controls. These alterations in lung progenitor function may be critical in the epithelial repair process.

This work reveals insights into the mechanisms of lung stem cell alveolar differentiation and provides opportunities for study of novel therapeutic opportunities to promote repair via stimulation of endogenous progenitor cells. We highlight the power of an *in vitro* system making it possible to study mechanisms underlying alterations in differentiation patterns. Together, these findings suggest defective supporting cell signaling to alveolar progenitors could occur in the setting of lung injury that can be rescued by MSC-secreted signals. Other organ systems have shown similar stimulation of stem cell functions by MSC. Bone marrow derived MSC have been shown to stimulate cardiac stem cell proliferation and differentiation^22^. MSC-mediated paracrine signaling to endogenous progenitors may be responsible for enhanced repair at sites of injury in multiple organ systems. It is also possible that other non-soluble factors produced by MSC, such as extracellular matrix, may also contribute to epithelial progenitor response and our findings.

We have shown using lineage tracing studies that CCSP-expressing cells, including club cells and distal lung epithelial cells, repair bleomycin-induced alveolar injury. Others have also shown that CCSP-positive cells give rise to lineage-labeled alveolar cells, including AT2 and AT1 cells, after bleomycin injury^1,14^. Yet, using the same lineage tracing mouse model, CCSP-expressing cells do not repair hyperoxia injury^4^ without any therapeutic factor administration. These contrasting results suggest that hyperoxia alters stem cell function, possibly due to altered supporting cell signaling, whereas bleomycin injury does not. MSC-secreted factors may replace the signals that hyperoxic injury impacts, triggering alveolar progenitor repair in the mouse model of BPD.

These studies show a direct effect of MSC-secreted factors on lung progenitor cell function. We previously defined a BMP4-NFATc1-TSP1 axis regulating adult lung epithelial progenitor cell differentiation and alveolar injury repair^10^. Lung injury and lung endothelial cell damage may lead to altered BMP4-NFATc1-TSP1 signaling and defective triggering of alveolar progenitor function. MSC-secreted factors such as TSP1 may replace missing or aberrant vascular signals and activate epithelial repair. The presence of TSP1 in MSC supernatant, together with our previous findings, suggests TSP1 is one way that MSCs can affect alveolar differentiation. MSC-secreted TSP1 has also been shown to rescue neuron synaptic dysfunction in a mouse model of Alzheimer’s disease^23^. Additional candidate MSC-secreted factors include ICAM, which is known to be induced during alveolarization^24^ and elevated after AT2 cell damage in rat injury models^25^, MMP-9, which can be elevated and dysregulated in lung disease^26^; and Epigen, which is altered in lung cancer suggesting a role in stem cell proliferation^27^. Additional work is necessary to further examine the contribution of these candidate secreted components.

MSC-secreted factors may replace missing or aberrant signals and activate epithelial repair. The 3D lung organoid assays employed allow a unique opportunity to study mechanisms of directing differentiation in alveolar progenitor populations. This assay could be used to test and identify therapeutic key soluble and non-soluble factors on candidate lung progenitor populations throughout the lung in response to specific lung injuries. There is significant translational relevance to these findings. Clinical trials are already underway using MSC in patients for which no real options exist for therapy or prevention. Recent phase 2a clinical trials have shown safety of bone marrow derived MSC intravenous delivery in moderate to severe acute respiratory distress syndrome (ARDS)^28^. MSC microvesicles have been shown to have therapeutic effects in an *ex vivo* perfused human lung model of severe bacterial pneumonia^29^. At this stage, it is highly critical to understand the mechanisms of therapeutic action of these cells and their secreted products to ensure safety and efficacy. This work supports this mission as we begin to understand the mechanisms by which MSC stimulate progenitor cell activity and how these mechanisms may be utilized in the future to achieve therapeutic lung injury repair.

## Methods

### Mice

All mice work was approved by the Children Hospital Boston Animal Care and Use Committee, accredited by AAALAC, and was performed in accordance with relevant institutional and national guidelines and regulations. 8–10 week old transgenic β-actin-EGFP mice (C57BL/6)^30^ or β-actin-DsRed mice (B6.Cg-Tg(ACTB-dsRed^∗^MST)1Nagy/J)^31^ were used for isolating lung epithelial cells. 2- to 4-week-old C57BL/6 J were used to isolate endothelial cells.

### Endothelial cell preparation

Lung MEC were isolated from 2- to 4-week-old mice by negative selection with anti-CD45-conjugated magnetic beads and positive selection with anti-CD31-conjugated magnetic beads. CD31-positive cells were then amplified in a gelatin-coated culture plate for 3–5 days followed by reselection with anti-CD31-conjugated magnetic beads. Endothelial cell purity was determined by IF staining for CD31, VE-Cadherin, and VEGFR2, and cells were used for experiments between passages 2 and 6. For Ac-LDL uptake, endothelial cells were incubated with 10 μg/ml Dil-ac-LDL for 2 hr at 37 °C followed by staining with DAPI. For matrigel tube formation, endothelial cells were seeded on matrigel-coated 24-well plates (1 × 10^5^ cells/well), incubated for 1 hr at 37 °C, and added fresh medium. One to 3 days after plating, tube formation was confirmed.

### MSC preparation

MSC were provided by the Hershenson laboratory. Bone marrow MSC were isolated and tested in the Prockop laboratory. MSC were isolated from femurs or lungs of mice (BALB/cByJ, Jackson Labs, Bar Harbor, ME) and flow cytometry and differentiation analysis were performed and confirmed MSC properties. For bone-marrow derived MSC, as described by Peister^31^, cells were isolated from femurs, plated, and expanded per protocol previously described. Differentiation assays for osteogenesis, adipogenesis, and chondrocyte differentiation were performed^32^.

### Epithelial cell isolation and 3D organoid assay

Primary lung epithelial cells were isolated from 8–10 week old β-actin-EGFP mice or β-actin-dsRed mice, as previously described^10^ using pan CD45-APC, CD31-APC, Sca-1 (Ly-6A/E)-APC-Cy7 (PharMingen), EpCAM-PE-Cy7 (BioLegend) with 4′, 6-diamidino-2-phenylindole (DAPI) (Sigma) staining to eliminate dead cells. Cell sorting was performed with a BD FACS Aria and data were analyzed with FlowJo software (Tree Star, Inc.). Amplified MEC or MSC were used for 3D co-cultures of Epcam^+^ Sca-1^−^ cells and Epcam^+^ Sca-1^+^ cells or dissociated organoids from the latter. MEC or MSC were added to growth factor–reduced Matrigel (BD Bioscience) at 1 × 10^6^ cells/ml. Sorted Epcam^+^ Sca-1^+^ and Epcam^+^ Sca-1^−^ cells were resuspended in the MEC or MSC containing Matrigel, which was prediluted at a ratio of 1:1 with medium and added to a 24-well transwell filter inserts with 0.4um pore (Corning) in a 24-well tissue culture plate containing 500 μl of medium. Dulbecco’s Modified Eagle’s Medium/F12 (Invitrogen) was supplemented with 10% FBS, penicillin/streptomycin, 1 mM HEPES, and insulin/transferrin/selenium (Sigma) for all cultures. Cultures were incubated at 37 °C in a humidified incubator (5% CO_2_), and the medium was replaced every other day for up to 14 days. For serial passages, Epcam^+^ Sca-1^+^ and Epcam^+^ Sca-1^−^ cells 3D cocultures were dissociated in dispase (BD Bioscience) and trypsin (GIBCO) to generate a single-cell suspension followed by FACS for GFP (or DsRED). GFP^+^ (or DsRED^+^) cells were resuspended with fresh MEC or MSC/Matrigel mixtures. Typically, 2,000 cells were plated for primary organoid (1°) formation. Primary organoids were dissociated, FACS sorted for GFP (or DsRED), and 2,500 GFP^+^ (or DsRED^+^) cells were replated with fresh MEC or MSC/Matrigel mixture for secondary (2°) and subsequent (3° and 4°) organoid formation biweekly.

### Conditioned media preparation

MEC and MSC confluent cultures were incubated in serum-free α-MEM media for 24 hours, and conditioned media representing equal number of cells were concentrated 10-fold or 5-fold using Amicon Ultra Centrifugal Filter Device (Millipore, Billerica, MA) with a molecular weight cutoff of 10 kDa as per manufacturer’s recommendations. 1x, 0.1x conditioned media and non-conditioned medium was also prepared with serum-free α-MEM.

### RNA isolation, reverse transcription and quantitative real-time PCR

Total RNA was isolated from 3D culture wells using Absolutely RNA Nanoprep Kit (Agilent). cDNA was synthesized from total RNA using the Superscript III RT (Invitrogen). PCRs were performed using a Taqman Gene Expression Master Mix (Invitrogen) with specific taqman probes for Gapdh (Invitrogen 4352339E) and Sftpc (Invitrogen Mm00488144_m1).

### Histology and immunofluorescence

Paraffin sections were stained with hematoxylin/eosin (H&E) (Sigma) or immunostained. For immunofluorescence, after deparaffinazion and antigen retrieval process, sections were incubated with primary antibodies according to manufacturer’s instructions after blocking for 1 h at room temperature. The slides were then washed and incubated with the appropriate secondary antibodies and labeling dyes. Secondary antibodies were coupled to Alexa-488, or Alexa-568 fluorochromes. After washing, tissue sections were mounted with Prolong gold with DAPI. Primary antibodies used are the following: SPC (Abcam ab211326), CCSP (Santa Cruz sc-390313), Podoplanin (Abcam ab11936) and Aquaporin 5 (Abcam ab11936).

### Antibody array and western blotting analysis

The mouse-specific biotin label-based antibody array (RayBio® Biotin Label-based Mouse Antibody Array I, L-Series, RayBiotech, Norcross, USA) that detects 308 different mouse target proteins was performed using MSC conditioned media. Two hundred *μ*g of supernatant protein were biotin- labeled according to manufacturer’s instructions. Biotin-labeled supernatant were incubated with the array overnight at 4 °C followed by hybridization with HRP-conjugated streptavidin. Mouse antibody arrays were developed using Pierce ECL2 kit (Pierce, Thermo scientific) and quantified with Typhoon 9410 scanner-ImageQuantTL 7.0 software (GE Healthcare Europe, Barcelona, Spain). The analysis was performed with RayBio® Antibody Array Analysis Tool using a normalization according to positive control densities after background subtraction.

For the Western Blotting, cell lysates from MEC and MSC were resolved in 15% polyacrylamide gels and electrotransferred. Membranes were probed with the following antibodies: Thrombospondin-1 (TSP1, Thermo Scientific MS-1066-P1ABX) and B-Actin (Abcam ab8226).

### Digital image acquisition and processing

Digital images were acquired using: 1) Nikon/eclipse TE2000-S equipped with a digital Sight DS2mvw camera and 2) Nikon/eclipse 90i equipped with a Nikon DSF11 camera. Acquisition was performed using NIS software. Images were composed and analyzed with Fiji software. To assess organoid size, individual colonies were manually outlined and their area was determined with Fiji.

### Statistical analysis

All experiments were carried out at least three times independently. Graph-Pad Prism (GraphPad Software, La Jolla, CA, USA) and Mstat (Mstat Software, McArdle Laboratory for Cancer Research, University of Wisconsin-Madison, Madison, WI, USA) were used for graphing and statistical analyses. Kruskal-Wallis test (independent samples, two-sided) was used for pairwise comparisons among groups at each condition. More than two groups of data were analyzed by one-way ANOVA with non-parametric Kruskal-Wallis test. In the figures, ^***^P ≤ 0.001, ^**^P ≤ 0.01, and ^*^P ≤ 0.05.

## Supplementary information

Supplementary Info

## Data Availability

All data generated or analyzed during this study are included in this published article (and its Supplementary Information files) or available from the corresponding author on reasonable request.
