# Supplementary material for: Mesenchymal Stem Cells Increase Alveolar Differentiation in Lung Progenitor Organoid Cultures
Source: Sci Rep. 2019 Apr 23;9:6479. doi: 10.1038/s41598-019-42819-1 (PMC6478947; doi:10.1038/s41598-019-42819-1)
Supplement: Supplementary file 1 — Supplementary Info [file 41598_2019_42819_MOESM1_ESM.pdf]

Title: Mesenchymal Stem Cells Increase Alveolar Differentiation in Lung Progenitor Organoid Cultures

Authors: Kristen T. Leeman, MD*[1], Patrizia Pessina, PhD[2,3,4], Joo-Hyeon Lee, PhD[2,3,4,5], Carla F. Kim, PhD*[2,3,4]

Affiliations: [1]Department of Pediatrics, Division of Newborn Medicine; Boston Children's Hospital; Boston, MA, 02115; USA; [2]Department of Medicine, Division of Hematology/Oncology; Stem Cell Program; Boston Children's Hospital; Boston, MA, 02115; USA; [3]Genetics Department, Harvard Medical School, Boston, MA 02115; USA; [4]Harvard Stem Cell Institute, Cambridge, MA 02138; USA; [5]Wellcome Trust/Medical Research Council Stem Cell Institute, University of Cambridge, Tennis Court Road, Cambridge CB2 1QR, UK

Supplementary Figure 1.

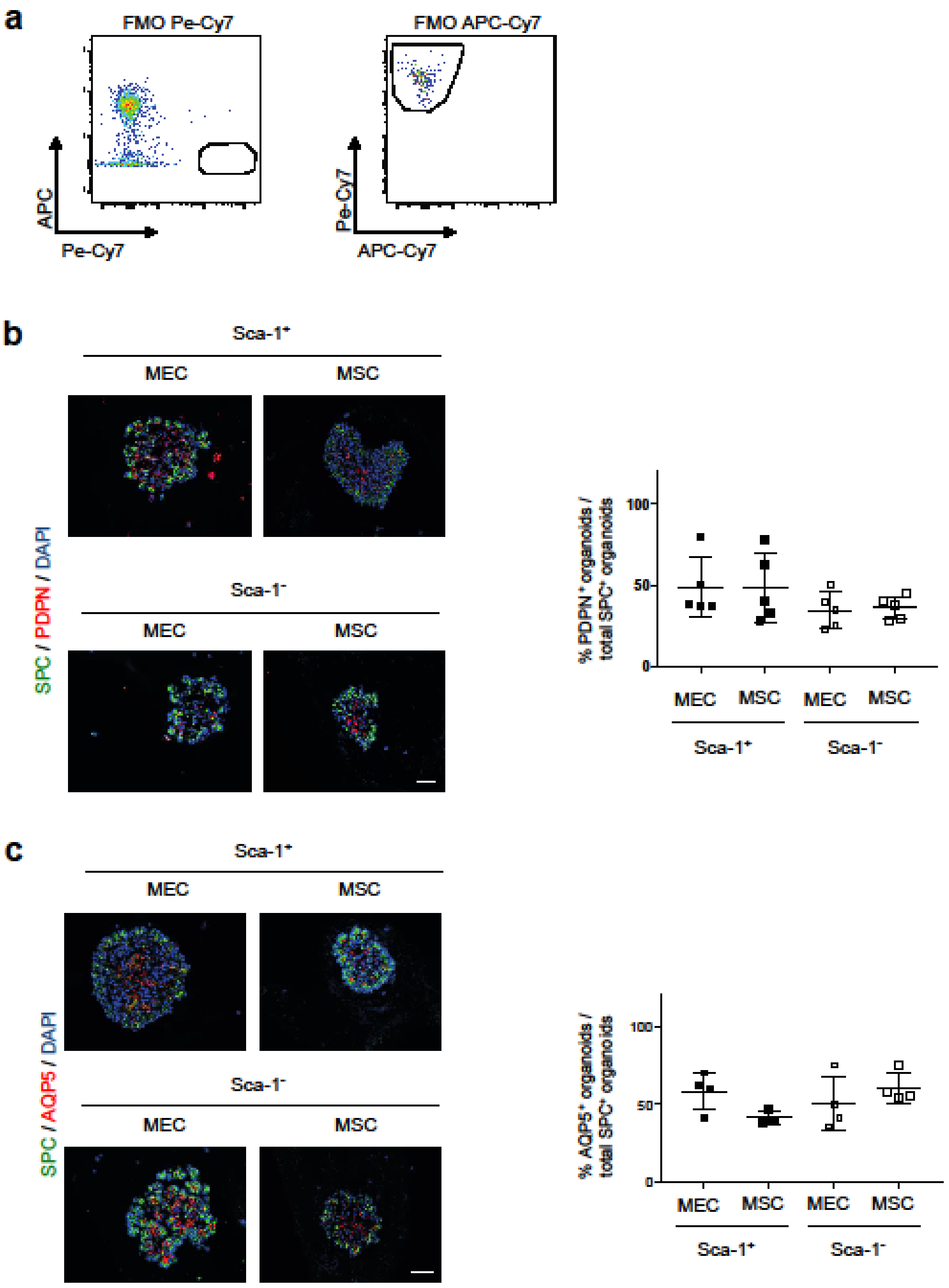

Supplementary Figure 1. Mesenchymal Stem Cells Have No Significant Effect on AT2 to AT1 Differentiation in 3D Co-Culture.

(**a**) PE-Cy7 (EpCam) and APC-Cy7 (Sca-1) FMO (Fluorescence Minus One) controls of the epithelial cell FACS isolation strategy are shown.

(**b**) Left panel: Representative images of alveolar organoids from MEC (left) and MSC (right) stained with SPC (green) and Podoplanin (PDPN, red) antibodies. Nuclei are stained with DAPI (blue). Scale bar: 50μM. Right panel: percentage of $PDPN^+$ organoids of the alveolar (SPC^+) organoids derived from Sca-1^+ and Sca-1^- cells cultured with the two stromal cell populations (MEC and MSC). Data presented are the mean of five independent experiments with triplicate wells. Error bars represent SD. (**c**) Left panel: Representative images of alveolar organoids from MEC (left) and MSC right) stained with SPC (green) and Aquaporin 5 (AQP5, red) antibodies. Nuclei are stained with DAPI (blue). Scale bar: 50μM. Right panel: percentage of $AQP5^+$ organoids of the alveolar (SPC^+) organoids derived from Sca-1^+ and Sca-1^- cells cultured with the two stromal cell populations (MEC, and MSC). Data presented are the mean of four independent experiments with triplicate wells. Error bars represent SD.

Supplementary Figure 2.

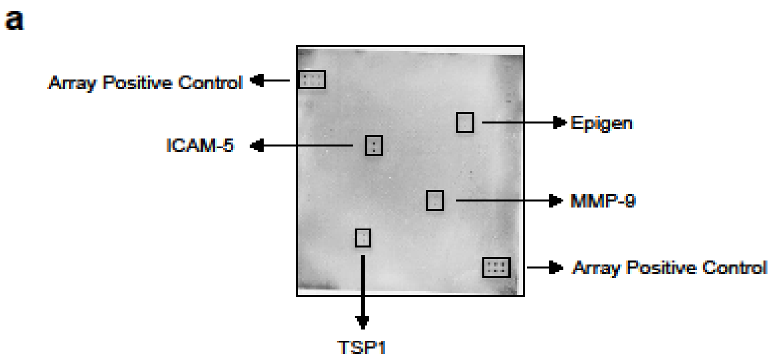

Supplementary Figure 2: Antibody Array on MSC Supernatant Identifies Potential Factors Involved In Lung Progenitor Cell Differentiation.

Antibody Array performed on MSC supernatant. Squares highlight proteins related to alveolarization and lung damage. Upper left and lower right squares indicate positive controls.
